# Impact of Fasting Blood Glucose Levels on Blood Pressure Parameters among Older Adults with Prediabetes

**DOI:** 10.1155/2023/1778371

**Published:** 2023-03-09

**Authors:** Thapanee Roengrit, Ruchada Sri-Amad, Nawiya Huipao, Suphawadee Phababpha, Piyapong Prasertsri

**Affiliations:** ^1^Department of Basic Medical Science, Faculty of Medicine Vajira Hospital, Navamindradhiraj University, Bangkok, Thailand; ^2^Department of Physical Therapy, Faculty of Allied Health Sciences, Chulalongkorn University, Bangkok, Thailand; ^3^Division of Health and Applied Sciences, Faculty of Science, Prince of Songkla University, Songkhla, Thailand; ^4^Chulabhorn International College of Medicine, Thammasat University, Pathum Thani, Thailand; ^5^Faculty of Allied Health Sciences, Burapha University, Saen Suk, Chonburi, Thailand; ^6^Exercise and Nutrition Innovation and Sciences Research Unit, Burapha University, Saen Suk, Chonburi, Thailand

## Abstract

Prediabetes mellitus (pre-DM) is defined as blood glucose levels higher than normal but lower than the threshold for diabetes mellitus (DM) diagnosis. Four-limb blood pressure (BP) differences can help identify a significant risk for cardiovascular diseases (CVDs). The current study aimed to determine the importance of BP and the ankle-brachial index (ABI) between two patient groups and the association between fasting blood glucose (FBG) levels and four-limb BP, ABI, interarm BP difference (IAD), and interleg BP difference (ILD). Moreover, the effect of cardiovascular risk factors on the development of pre-DM among older adults was evaluated. The participants were divided into the normal fasting glucose (NFG) and impaired fasting glucose (IFG) groups. Data on physical characteristics, lipid profiles, four-limb BP, ABI, IAD, ILD, and cardiovascular risk factors were assessed. The IFG group had a significantly higher systolic blood pressure (SBP) and pulse pressure than the NFG group (*p*  <  0.05). SBP was significantly positively correlated with FBG levels (*p*  <  0.05). The IFG group had a lower ABI than the NFG group (*p*  <  0.05). However, there was no significant difference in terms of IAD and ILD between the two groups. Furthermore, hypertension (HT), metabolic syndrome (MetS), and dyslipidemia were significantly correlated with a high prevalence of prediabetes (*p*  <  0.05). Individuals with prediabetes had a higher BP than those with normoglycemia. Prediabetes was correlated with HT, MetS, and dyslipidemia.

## 1. Introduction

Prediabetes mellitus (pre-DM) is defined as blood glucose (BG) levels higher than normal but lower than the threshold for the diagnosis of DM. It is well known that the prevalence of pre-DM rises with advancing age. Pre-DM is correlated with pathophysiological processes such as insulin resistance and/or defective insulin secretion [1]. The coexistence of high BG levels and high blood pressure (BP) is a risk factor of cardiovascular diseases (CVDs). Increased BP variability is correlated with the prevalence of pre-DM [2]. In addition, pre-DM is strongly correlated with high BP [2]. Several mechanisms could explain the effect of high BG levels on vascular constriction and, ultimately, increased BP. The mean diastolic BP (DBP) of young adults in the prediabetic group was significantly higher than that of young adults in the normoglycemic group. However, systolic BP (SBP) did not significantly differ between the two groups [3]. The influence of hyperglycemia and BP on vascular disease and mortality is age specific.

One-arm BP measurement is widely practiced. However, with this method, any underlying arterial disease can be missed out in some cases. Hence, four-limb BP differences are understandable. Presently, modern technology allows simultaneous BP measurement in all four limbs, which can provide a comprehensive assessment of BP and produce an accurate estimate of BP differences between the four limbs [4]. A previous study revealed that the initial screening of interarm BP differences (IADs) can identify significant risk factors for vascular diseases [5].

IAD, interleg BP differences (ILDs), and an ankle-brachial index (ABI) of <0.9 were correlated with peripheral artery disease (PAD) [6, 7]. PAD, which refers to the restriction of blood flow in the lower extremities, is linked to morbidity and mortality from other CVDs, particularly in older adults. Moreover, an IAD of >10 mm Hg and an ILD of >15 mm Hg were correlated with cardiovascular events [7–9], including increased cardiovascular mortality and all-cause mortality. Hence, the early detection of IAD and ILD, which can warrant further CVD assessment, and the management of CVD risk factors are considered important [5]. However, the importance of BP parameters in identifying early-stage PADs was challenging to evaluate. Furthermore, asymptomatic diabetes is not detected until the symptoms of advanced-stage limb ischemia such as ulcers and gangrene develop [10].

Therefore, the importance of BP parameters in older adults without DM has been controversial, and the number of studies on this topic is limited. There remains a gap regarding our understanding of the association between four-limb BP and BG levels. Hence, the current study aimed to assess the importance of BP and ABI between the normal fasting glucose (NFG) and impaired fasting glucose (IFG) groups and the association between fasting BG (FBG) levels and four-limb BP, ABI, IAD, and ILD. Moreover, the effect of cardiovascular risk factors, such as sex, obesity, hypertension (HT), metabolic syndrome (MetS), and dyslipidemia, on the development of pre-DM among older adults was identified. This research can provide further evidence for the appropriate prevention and control of the risk factors of vascular events and their outcomes.

## 2. Materials and Methods

### 2.1. Study Population

All participants were screened from February 2017 to March 2018 at Hat Yai Chivasuk's Health Promotion Center. The trained researchers included participants aged ≥60 years based on their medical records. In addition, some participants did not present with DM (an FBG level of < 126 mg/dL) for 3 months before the start of the study. However, participants with at least one significant medical history, such as CVD, stroke, DM, nephritis, and use of antihyperglycemic and antihypertensive medications, were excluded. Finally, 98 participants were included in the study, and they were divided into the NFG (*n* = 61) and IFG (*n* = 37) groups. Before participating in any of the study procedures, all participants provided written informed consent according to the general recommendations of the Declaration of Helsinki. This study was approved by the Research Ethics Committees of the Faculty of Medicine of the Prince of Songkla University (REC 60-166-19-2).

### 2.2. Data Collection

All participants were admitted to the health promotion center in the morning after overnight fasting. Data on the demographic and clinical characteristics of the participants were obtained from the medical records or via interviews with the participants. Body mass and height were measured while the participants were wearing a light dress without shoes. The body mass index (BMI) was calculated as weight (kg)/height (m^2^). Waist circumference (WC) was measured at the narrowest level between the lowest rib and the iliac crest. Hip circumference was measured at the widest portion of the buttocks using a standard tape measure in the standing position. Then, the waist-to-hip ratio was calculated.

Blood samples were drawn after overnight fasting (>12 h) to measure serum total cholesterol (TC), low-density lipoprotein cholesterol (LDL-C), high-density lipoprotein cholesterol (HDL-C) and triglyceride (TG) levels. FBG samples were collected from the cutaneous vein of the fingertip using a puncture device and a glucose meter (Roche, Almere, the Netherlands).

### 2.3. BP Parameters

Four-limb BP and ABI were measured using VaSera VS-1500 (Fukuda Denshi, Tokyo, Japan) according to the manufacturer's instructions. Both the simultaneous (all four limbs) and sequential (right limb followed by the left limb) settings for BP readings were used. The trained researchers placed the pressure cuff approximately 2 cm above the antecubital fossa on the arms and approximately 2 cm above the medial malleolus on the ankles. Then, the measurements were performed after the participants took a rest for approximately 10 min in the supine position. SBP and DBP were measured using an appropriate cuff. The pulse pressure (PP) was defined as the difference between SBP and DBP. The mean arterial pressure was two-third DBP plus one-third SBP. Subsequently, ABI was calculated using the ratio of the ankle SBP divided by the arm SBP. Data were automatically analyzed using the Vasava Data Management software program [11]. After obtaining the four-limb BP, IAD was calculated, and ILD was defined as the difference in bilateral limb SBPs [7].

### 2.4. Definitions

Participants with an FBG level of 100–125 and <100 mg/dL, according to the American Diabetes Association criteria, were included in the IFG and NFG groups, respectively [12]. MetS was defined as at least three of the following components according to the National Cholesterol Education Program, Adult Treatment Panel III criteria: (i) WC of >90 cm for men and >85 cm for women, (ii) TG levels of >150 mg/dL and/or drug treatment for elevated TG levels, (iii) HDL-C levels of <40 mg/dL for men and <50 mg/dL for women, (iv) an SBP of >130 mm Hg and DBP of >85 mm Hg, and (v) FBG levels of >100 mg/dL [13]. Dyslipidemia was defined as a TC level of >200 mg/dL, a TG level of >150 mg/dL, an LDL-C level of >130 mg/dL, or an HDL-C level of <40 mg/dL for men and HDL-C level of <50 mg/dL for women [14]. Obesity was defined as a BMI of  ≥30 kg/m^2^ based on the World Health Organization criteria. HT was defined as an SBP of >140 mm Hg or DBP of >90 mm Hg according to the JNC-7 criteria [15].

### 2.5. Statistical Analysis

All statistical analyses were performed using the Statistical Package for the Social Sciences software. Continuous variables with normal distribution were expressed as a mean ± standard deviation and those with nonnormal distribution as a median (interquartile range). Categorical variables were expressed as numbers (%). Differences in the means of categorical variables between the two groups were analyzed using the chi-square test. Meanwhile, the independent sample *t*-test or the Mann–Whitney *U* test was used to compare continuous variables based on distribution between the two groups. The correlation between FBG and BP parameters was analyzed using the Pearson correlation coefficients. Binary logistic regression with univariate analyses was performed on the models using pre-DM as a dichotomous-dependent variable (0 = absence of pre-DM and 1 = presence of pre-DM) and female sex, obesity, HT, MetS, and dyslipidemia as binary independent variables (0 = no and 1 = yes). A *p* value of <0.05 was considered statistically significant.

## 3. Results and Discussion

The participants in the IFG group were significantly older than those in the NFG group (*p*=0.02). The IFG group had higher TC and LDL-C levels than the NFG group (246.9 ± 44.5 vs. 221.4 ± 39.3 mg/dL and 170.7 ± 39.9 vs. 148.1 ± 39.7 mg/dL, respectively; *p*  <  0.01). However, the TG and HDL-C levels did not significantly differ between the two groups. There was no significant difference in the prevalence of dyslipidemia, HT, and MetS between the two groups (Table 1).

According to BP and ABI, the IFG group had a significantly higher SBP than the NFG group (right arm: 135.7 ± 16.8 vs. 125.8 ± 14.3 mm Hg, *p*  <  0.01; left arm: 135.3 ± 15.9 vs. 129.1 ± 15.1 mm Hg, *p*  <  0.01; right ankle: 151.0 ± 17.0 vs. 142.1 ± 18.4 mm Hg, *p*=0.02; and left ankle: 152.4 ± 16.6 vs. 147.4 ± 16.7 mm Hg, *p*=0.02, respectively). The NFG group had a higher PP than the IFG group (right arm: 55 (35, 100) vs. 49 (33, 75), *p*=0.01; left arm: 54.7 ± 12.5 vs. 50.3 ± 11.4 mm Hg, *p*  <  0.01; right ankle: 73.8 ± 14.2 vs. 66.8 ± 14.2 mm Hg. *p*=0.02; and left ankle: 73.3 ± 13.9 vs. 69.5 ± 13.4 mm Hg, *p*=0.03, respectively). The IFG group had a lower ABI in both sides than the NFG group (right limb: 1.1 (0.8, 1.2) vs. 1.1 (0.7, 1.8), *p*=0.02; left limb: 1.1 (0.9, 1.2) vs. 1.1 (0.8, 1.7), *p*=0.04). However, IADs and ILDs did not significantly differ between the two groups (Table 2).

FBG levels were significantly positively correlated with SBP (right arm: *r* = 0.23, *p*=0.02; left arm: *r* = 0.22, *p*=0.02; and right ankle: *r* = 0.22, *p*=0.02) and PP (right arm: *r* = 0.20, *p*=0.04; left arm: *r* = 0.20, *p*=0.04; and right ankle: *r* = 0.24, *p*=0.01). However, there were no significant correlations between FBG levels and DBP, MAP, ABI, IAD, and ILD (Table 3).

Based on regression analyses, HT, MetS, and dyslipidemia were significantly correlated with a higher prevalence of pre-DM (unadjusted odds ratios (OR): 3.03, *p*=0.03; OR: 4.13, *p*=0.01; and OR: 0.12, *p*=0.01, respectively). However, female sex and obesity did not affect the prevalence of pre-DM (Table 4).

The current study investigated the impact of FBG levels on BP parameters including SBP, DBP, PP, MAP, IAD, ILD, and ABI. Results showed that individuals with pre-DM had significantly higher SBP and PP levels than those with normoglycemia. A positive correlation was observed between FBG and SBP levels. However, the association between FBG and DBP levels was not significant. These findings are consistent with those of a cross-sectional study with Northeast Chinese adults aged 18–79 years. Results showed that FBG levels were positively associated with SBP in high quantiles in both men and women after adjusting for age, BMI, WC, smoking status, alcohol consumption, and dyslipidemia [16]. Conversely, in another research, young adults with pre-DM had a significantly higher DBP level than those with normoglycemia in India. Nevertheless, there was no significant difference in terms of SBP levels [4]. Moreover, the IFG group had a significantly higher four-limb PP than the NFG group. PP represents the pulsatile component of blood flow. Increased PP is associated with impaired coronary flow reserve, which results in microvascular dysfunction [17]. However, studies comparing differences in BG and PP levels in individuals without DM are limited. Several potential mechanisms could explain the deleterious effect of hyperglycemia on the arteries, leading to high vascular resistance and, ultimately, increased BP. First, hyperglycemia activates the renin-angiotensin system [16, 18] and induces angiotensinogen transcription and angiotensin II production. Angiotensin II promotes the proliferation and migration of smooth muscle cells and stimulates collagen synthesis leading to decreased vascular diameter [19]. Second, hyperglycemia is closely correlated with increased sympathetic nervous system activity, which contributes to the development of high arterial BP. Sympathetic nervous system activity is strongly associated with hyperglycemia. In addition, insulin resistance and HT are strongly associated with sympathovagal imbalance [20]. Therefore, both sympathetic nerve stimulation and renin-angiotensin system activation under hyperglycemic conditions contribute to sodium retention, which causes increased arterial BP. Third, hyperglycemia promotes the reaction between proteins or lipids and aldose sugars and increases the cross-linking of elastin, collagen, and other molecules commonly referred to as advanced glycation end products [21]. These compounds can trigger a series of responses involving oxidation and inflammation, which induce endothelial dysfunction by reducing the phosphorylation status and expression of endothelial nitric oxide synthase (eNOS), thereby contributing to the development of vascular complications [6].

Abnormal IAD and ILD were not clearly defined between the two groups in our study. The results might be attributed to the glycemia status in pre-DM, which is not high enough to alter vascular dysfunction. The association between FBG and BP levels became stronger when BG levels reached 5.6 mmol/L [16]. However, IAD can be evaluated when assessing new patients with type 2 DM [8, 22, 23]. The presence of IAD was defined as a difference in a systolic BP of ≥10 mm Hg in 10% of patients with DM. A larger IAD may imply vascular blockage due to atherosclerosis in older adults [5, 24, 25], and this is associated with the presence of PAD.

An abnormal ABI has a high specificity for predicting future cardiovascular events [26]. The current study showed that the IFG group had a lower ABI in both sides than the NFG group. A clinical cutoff point of ABI for the diagnosis of PAD is less than 0.9. Thus, values shown in our results may not be clinically relevant [6, 7]. However, the cutoff point was obtained from patients with DM who have higher glycemic data than our participants. In addition, our findings also provide important evidence that individuals with pre-DM are at an increased risk for PAD and related CVDs [26]. In individuals with type 2 DM, a recent investigation revealed a correlation between ABI and FBG levels [27]. The progression of glycemic status leads to vascular insufficiency, which increases the risk of PAD. Hence, the early detection of PAD in patients with pre-DM is important [26].

In our study, with consideration of the differences in lipid levels, the NFG group had significantly higher TC and LDL-C levels than the IFG group. In addition, dyslipidemia was significantly associated with a prevalence of pre-DM. This finding is consistent with those of recent studies investigating the association between pre-DM and lipid metabolism disorders [28]. The prevalence of dyslipidemia was 95% in patients with hyperglycemia [29]. Lipid profile changes lead to elevated free fatty acid levels, which may induce insulin resistance and *β*-cell dysfunction [30]. High LDL-C levels can result in the accumulation of TG in the vessels and the development of atherosclerosis, which is a cardiovascular complication [20]. The TC level is commonly normal or near-normal if glycemic control is adequate, and it increases if glucose levels are not well controlled [31]. The difference in findings may be correlated with lifestyle modification [32].

Our results were in accordance with those of previous studies supporting the notion that pre-DM was a risk factor for HT. Compared with NFG, IFG is associated with 1.81 times higher risk for HT in elderly Chinese individuals. Higher FBG levels within the normal range were significantly correlated with a higher risk of HT in both sexes compared with lower FBG levels after adjusting for age, smoking, and alcohol consumption [33]. In elderly Chinese participants, elevated FBG levels within the normal range were associated with a higher risk of developing HT during a 5-year follow-up after adjusting for age and sex [34]. The presence of IFG plays an important role in determining CVD-related mortality associated with moderate systolic HT if SBP is >140 mm Hg [34]. Therefore, patients with pre-DM who had an SBP of 120–130 mm Hg had the lowest CVD-related mortality risk. The optimal SBP range associated with the lowest mortality was gradually higher with worsening glucose levels [35]. Moreover, the results showed that MetS could influence the prevalence of pre-DM. According to a recent study, MetS was relatively common in patients with pre-DM as evidenced by a higher number of MetS diagnostic criteria and a greater risk of pre-DM [12]. However, whether pre-DM or IFG alone in the absence of HT, dyslipidemia, MetS, or their combination is associated with an increased risk for CVD remains unclear. Some studies did not completely identify concomitant cardiovascular risk factors. Hence, these findings should be interpreted with caution [36].

This cross-sectional study had several limitations. First, participants were from a single center within a specific period, and only a small sample size was studied. The findings may not be representative of individuals from other areas. Second, using univariate logistic regression analyses due to the small sample size provides unadjusted OR that is unknown whether HT, MetS, and dyslipidemia were independently associated with IFG. Third, some patients lacked information such as medications used, level of physical activity, and dietary intake. Lastly, the oral glucose tolerance test or HbA1c measurement was not performed in this study, which might have led to a bias in estimating glycemic status and confirming pre-DM condition. The prevalence of pre-DM and DM was higher when HbA1c levels rather than FPG levels were evaluated [37].

## 4. Conclusions

Individuals with elevated BG levels but within the normal range had a higher BP than those with normoglycemia. IFG was correlated with HT, MetS, and dyslipidemia. Hence, initial BP monitoring can identify vascular abnormalities in individuals with pre-DM and DM who are at risk for CVD and mortality. Furthermore, modification of lifestyle habits such as physical activity and diet can control BG levels.

## Figures and Tables

**Table 1:** Demographic, anthropometric, and biochemical parameters of the study population.

Parameters	Total (*n* = 98)	NFG group (*n* = 61)	IFG group (*n* = 37)	*p* value
Age (years)	65 (60, 76)	64 (60, 75)	68 (60, 76)	0.02^a^
Female, no. (%)	80 (81.6)	49 (80.3)	31 (83.7)	0.79
RHR (beats/min)	68.8 ± 8.9	68.5 ± 9.6	69.3 ± 7.7	0.68
Height (cm)	1.5 ± 0.0	154.4 ± 20.9	155.6 ± 8.7	0.38
Body mass (kg)	59.6 ± 7.6	59.9 ± 7.4	59.1 ± 7.9	0.60
BMI (kg/m^2^)	24.1 (18.2, 36.2)	24.3 (18.2, 29.7)	23.4 (18.7, 36.2)	0.33
WC (cm)	85.9 ± 8.3	86.8 ± 7.6	84.4 ± 9.1	0.21
HC (cm)	96.5 (84, 120)	96 (84, 120)	98 (85, 111)	0.60
WHR	0.8 ± 0.0	0.8 ± 0.0	0.8 ± 0.0	0.26
FBG (mg/dL)	98.7 ± 8.6	93.4 ± 4.2	107.5 ± 6.5	<0.01^b^
TC (mg/dL)	237.3 ± 44.2	221.4 ± 39.5	246.9 ± 44.5	<0.01^b^
HDL-C (mg/dL)	61 (25, 110)	61 (25, 100)	61 (41, 110)	0.37
TG (mg/dL)	86 (30, 268)	97 (30, 247)	81 (41, 268)	0.26
LDL-C (mg/dL)	162.2 ± 41.1	148.1 ± 39.7	170.7 ± 39.9	<0.01^b^
Dyslipidemia, no. (%)	85 (86.7)	46 (75.4)	34 (91.8)	0.02^a^
HT, no. (%)	26 (26.5)	11 (18.0)	15 (40.5)	0.01^a^
MetS, no. (%)	19 (19.3)	7 (11.4)	12 (32.4)	0.01^a^
Obesity, no. (%)	34 (34.6)	23 (37.7)	11 (29.7)	0.51

^a^ *p*  <  0.05; ^b^*p*  <  0.01. Continuous data were described as a mean ± SD and median (interquartile range). Categorical data were described as numbers (%). The chi-square test, independent sample *t*-test, and Mann–Whitney *U* test were used where appropriate. NFG = normal fasting glucose; IFG = impaired fasting glucose; RHR = resting heart rate; BMI = body mass index; WC = waist circumference; HC = hip circumference; WHR = waist-to-hip ratio; FBG = fasting blood glucose; TC = total cholesterol; HDL-C = high-density lipoprotein cholesterol; TG = triglyceride; LDL-C = low-density lipoprotein cholesterol; HT = hypertension; MetS = metabolic syndrome.

**Table 2:** BP parameters in the study population.

Parameters	Total (*n* = 98)	NFG group (*n* = 61)	IFG group (*n* = 37)	*p* value
*Right arm BP (mm Hg)*				
SBP	129.6 ± 15.9	125.8 ± 14.3	135.7 ± 16.8	<0.01^b^
DBP	76 (57, 100)	75 (57, 100)	80 (65, 94)	0.03^a^
PP	51 (33, 100)	49 (33, 75)	55 (35, 100)	0.01^a^
MAP	95 (74, 125)	91 (74, 125)	98 (78, 115)	0.11

*Left arm BP (mm Hg)*				
SBP	125.3 ± 13.5	129.1 ± 15.1	135.3 ± 15.9	<0.01^b^
DBP	77.7 ± 9.4	78.7 ± 9.3	80.5 ± 9.0	0.14
PP	47.6 ± 9.8	50.3 ± 11.4	54.7 ± 12.5	<0.01^b^
MAP	93.5 ± 9.9	95.5 ± 10.3	98.8 ± 10.2	0.01^a^
IAD (mm Hg)	5.2 ± 4.1	5.5 ± 3.9	4.3 ± 4.2	0.09
IAD >10 mm Hg, no. (%)	11 (11.2)	9 (14.7)	2 (5.4)	0.38

*Right ankle BP (mm Hg)*				
SBP	145.5 ± 18.3	142.1 ± 18.4	151.0 ± 17.0	0.02^a^
DBP	75 (63, 103)	74 (63, 103)	76 (66, 93)	0.15
PP	69.5 ± 14.5	66.8 ± 14.2	73.8 ± 14.2	0.02^a^
MAP	99.1 ± 10.2	97.5 ± 10.7	101.8 ± 9.1	0.04^a^

*Left ankle BP (mm Hg)*				
SBP	144.4 ± 16.1	147.4 ± 16.7	152.4 ± 16.6	0.02^a^
DBP	78 (63, 102)	78 (63, 102)	79 (63, 100)	0.31
PP	67.1 ± 12.6	69.5 ± 13.4	73.3 ± 13.9	0.03^a^
MAP	99.6 ± 9.9	101.1 ± 10.0	103.5 ± 9.8	0.07
ILD (mm Hg)	6.0 ± 5.5	6.6 ± 6.2	5.0 ± 4.0	0.17
ILD > 15 mm Hg, no. (%)	5 (5.1)	4 (6.5)	1 (2.7)	0.96
Right ABI	1.1 (0.7, 1.8)	1.13 (0.7, 1.8)	1.12 (0.8, 1.2)	0.02^a^
Left ABI	1.1 (0.8, 1.7)	1.13 (0.8, 1.7)	1.11 (0.9, 1.2)	0.04^a^
ABI < 0.9, no. (%)	3 (3.0)	2 (3.2)	1 (2.7)	0.80

^a^ *p*  <  0.05; ^b^*p*  <  0.01. Continuous data were described as a mean ± SD and median (interquartile range). Categorical data were described as numbers (%). The chi-square test, independent sample *t*-test, and Mann–Whitney *U* test were used where appropriate. NFG = normal fasting glucose; IFG = impaired fasting glucose; BP = blood pressure; SBP = systolic blood pressure; DBP = diastolic blood pressure; PP = pulse pressure; MAP = mean arterial pressure; IAD = interarm difference; ILD = interleg difference; ABI = ankle-brachial index.

**Table 3:** Correlation between FBG levels and BP parameters of the study population.

Parameters	Right limb		Left limb	
	Correlation coefficient (*r*)	*p* value	Correlation coefficient (*r*)	*p* value
*Arm BP (mm Hg)*				
SBP	0.23	0.02^a^	0.22	0.02^a^
DBP	0.15	0.12	0.11	0.24
PP	0.20	0.04^a^	0.20	0.04^a^
MAP	−0.16	0.10	0.18	0.06
IAD	−0.16	0.11		

*Ankle BP (mm Hg)*				
SBP	0.22	0.02^a^	0.17	0.08
DBP	0.08	0.41	0.07	0.48
PP	0.24	0.01^a^	0.17	0.08
MAP	0.18	0.07	0.13	0.18
ILD	−0.10	0.29		
ABI	−0.12	0.21	−0.12	0.20

^a^ *p*  <  0.05. *p* values were calculated using Pearson's correlation coefficient (*r*). FBG = fasting blood glucose; BP = blood pressure; ABI = ankle-brachial index; SBP = systolic blood pressure; DBP = diastolic blood pressure; PP = pulse pressure; MAP = mean arterial pressure; IAD = interarm difference; ILD = interleg difference.

**Table 4:** Binary logistic regression with univariate analyses to identify independent predictors of prediabetes.

Parameters	*β*	SE	OR (95% CI)	*p* value
Female sex	0.83	0.64	2.30 (0.65–8.16)	0.20
Obesity	−0.51	0.53	0.59 (0.21–1.69)	0.33
HT	1.11	0.53	3.03 (1.06–8.65)	0.03^a^
MetS	1.41	0.60	4.13 (1.26–13.48)	0.01^a^
Dyslipidemia	2.08	0.76	0.12 (0.02–0.55)	0.01^a^

^a^ *p*  <  0.05. *β* = coefficient for the constant; SE = standard error; OR = unadjusted odds ratios; CI = confidence interval; HT = hypertension, MetS = metabolic syndrome.

## Data Availability

The datasets generated and analyzed during the current study are available from the corresponding author on reasonable request.
